# Supplementary material for: Splice-Junction-Based Mapping of Alternative Isoforms in the Human Proteome
Source: Cell Rep. Author manuscript; Available in PMC 2020 Jan 15. (PMC6961840; doi:10.1016/j.celrep.2019.11.026)
Supplement: 2 [file NIHMS1546469-supplement-2.zip › DF1/PXD010154/Testis/Testis_11_CD99_GEEEQGEVDMESHR.pdf]

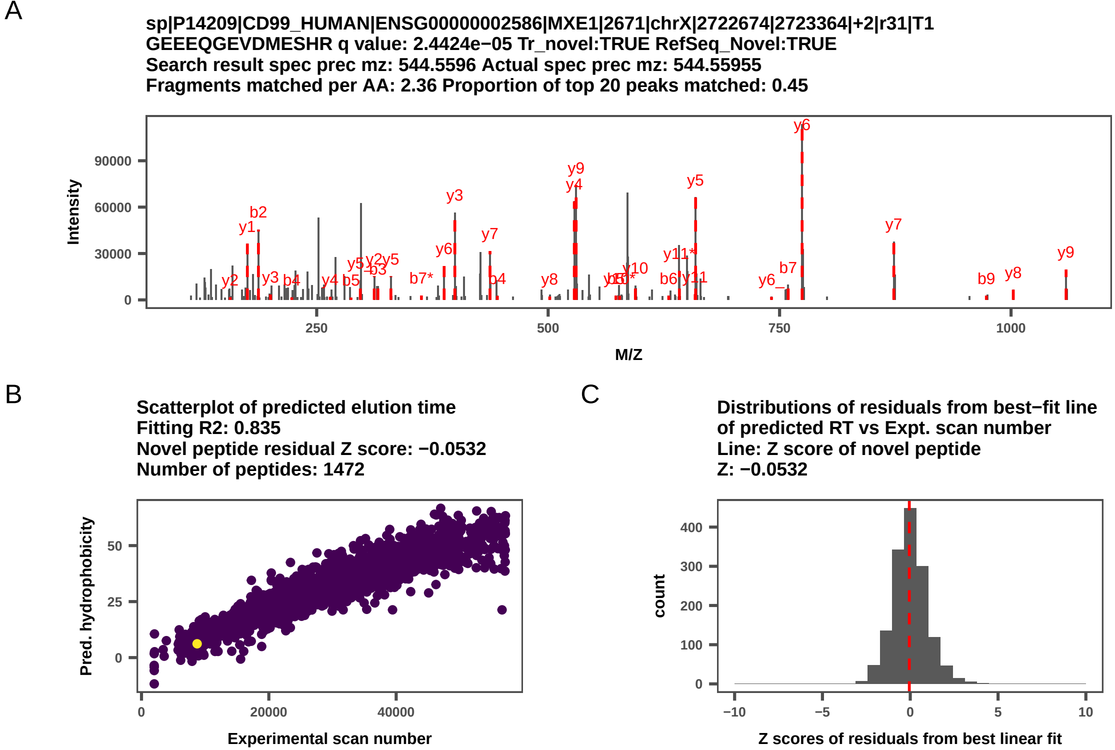
A
sp|P14209|CD99_HUMAN|ENSG00000002586|MXE1|2671|chrX|2722674|2723364|+2|r31|T1
GEEEQGEVDMESHR q value: 2.4424e−05 Tr_novel:TRUE RefSeq_Novel:TRUE
Search result spec prec mz: 544.5596 Actual spec prec mz: 544.55955
Fragments matched per AA: 2.36 Proportion of top 20 peaks matched: 0.45
Intensity
90000
60000
30000
0
250
500
750
1000
M/Z
y6
y9
y4
y5
y3
b2
y1
y7
y6
y7
y9
y2
y3
b4
y4
b5
b3
b7*
b4
y8
y10
b6
y11
y11*
b7
b9
y8
B
Scatterplot of predicted elution time
Fitting R2: 0.835
Novel peptide residual Z score: −0.0532
Number of peptides: 1472
Pred. hydrophobicity
50
25
0
0
20000
40000
Experimental scan number
C
Distributions of residuals from best−fit line
of predicted RT vs Expt. scan number
Line: Z score of novel peptide
Z: −0.0532
count
400
300
200
100
0
−10
−5
0
5
10
Z scores of residuals from best linear fit
